# A Spatial-Spectral Approach for Visualization of Vegetation Stress Resulting from Pipeline Leakage

**DOI:** 10.3390/s8063733

**Published:** 2008-06-04

**Authors:** Harald van der Werff, Mark van der Meijde, Fokke Jansma, Freek van der Meer, Gert Jan Groothuis

**Affiliations:** 1 International Institute for Geo-information Science and Earth Observation (ITC), Department of Earth Systems Analysis, P.O.Box 6, 7500 AA Enschede, The Netherlands; E-mail: vandermeijde@itc.nl; 2 DHV BV, P.O.Box 685, 9700 AR, Groningen, The Netherlands; 3 Utrecht University, Faculty of Geosciences. P.O.Box 80.021, 3508 TA, Utrecht, The Netherlands; 4 Nederlandse Aardolie Maatschappij B.V., P.O.Box 28.000, 9400 HH, Assen, The Netherlands

**Keywords:** pipeline, hydrocarbon, vegetation stress, hyperspectral, spatial

## Abstract

Hydrocarbon leakage into the environment has large economic and environmental impact. Traditional methods for investigating seepages and their resulting pollution, such as drilling, are destructive, time consuming and expensive. Remote sensing is an efficient tool that offers a non-destructive investigation method. Optical remote sensing has been extensively tested for exploration of onshore hydrocarbon reservoirs and detection of hydrocarbons at the Earth's surface. In this research, we investigate indirect manifestations of pipeline leakage by way of visualizing vegetation anomalies in airborne hyperspectral imagery. Agricultural land-use causes a heterogeneous landcover; variation in red edge position between fields was much larger than infield red edge position variation that could be related to hydrocarbon pollution. A moving and growing kernel procedure was developed to normalzie red edge values relative to values of neighbouring pixels to enhance pollution related anomalies in the image. Comparison of the spatial distribution of anomalies with geochemical data obtained by drilling showed that 8 out of 10 polluted sites were predicted correctly while 2 out of 30 sites that were predicted clean were actually polluted.

## 1 Introduction

In 1996, the Nederlandse Aardolie Maatschappij (NAM, Dutch Oil Company) constructed a 21 km long pipeline for transportation of benzene condensates from Grijpskerk to Anjum, respectively located in the provinces Groningen and Friesland in The Netherlands. Since its placement, the pipeline has started ‘sweating’ at connection points located at 9 m intervals along the pipeline. Extensive investigation along a 1 km trace revealed that approximately 50% of the connection points were sweating. The leaking of benzene condensate into the subsurface has large environmental and economical implications.

Traditional methods for investigating the presence of hydrocarbons, such as drilling and geochemical analysis, are time consuming, destructive and expensive. Remote sensing proved to be a tool that offers non-destructive investigation. Optical remote sensing has been extensively tested for detection of hydrocarbons at the Earth's surface [e.g. 1–4]. Theoretically, remote sensing is a suitable tool for direct and indirect detection of the presence of hydrocarbons in the near-subsurface. Oxidation of hydrocarbons at the surface leads to a wide range of geochemical anomalies in soil and vegetation. An overview of resulting anomalies and possibilities for remote sensing for indirect detection is given by [5–8].

Recently, numerous publications have been written on the use of imaging spectroscopy for the detection of botanical and mineralogical alterations resulting from natural gas seepage and man-induced hydrocarbon leakages [e.g. 6, 9–13]. Observed geochemical and botanical anomalies are usually subtle and not a unique indicator for the presence of hydrocarbons in the subsurface [7, 10, 12]. Changes in vegetation density appear much stronger in reflectance spectra than mineralogical anomalies that result from hydrocarbon presence [10]. Hydrocarbon pollution may influence vegetation directly, or indirectly by influencing the chemical environment of a soil [6, 7]. Although the influence of gases on vegetation density an plant vigor is shown [12, 13], it is not completely understood whether this effect is direct (e.g., poisoning) or indirect (e.g., lack of oxygen due to the presence of gases).

A recent investigation [9] showed the possibilities of field reflectance spectroscopy in detection of vegetation anomalies related to leakage of benzene condensate. The effect of benzene condensate, being a liquid, on plant vigor and crop performance is yet unknown. For this reason, spectral vegetation indices were correlated with drilling records which partly overlapped with field reflectance measurements [9]. Shift in red edge wavelength position [14] proved to be a suitable parameter for determining vegetation state in relation to the presence of benzene condensate. Pollution levels, estimated from drilling records, were generally in agreement with anomalous regions in the vegetation health. As they showed that field spectroscopy is capable of detecting botanical anomalies related to pipeline leakage, the use of airborne or space borne hyperspectral imagery is therefore promising.

The research presented in this paper builds on the previous study and aims to upscale the red edge indicator found in field spectral measurements to airborne hyperspectral imagery in order to map leakages along the 21 km extent of the NAM pipeline. The pipeline crosses an area with numerous agricultural fields. Due to natural variation between the various fields, we developed a normalization algorithm that allows for all fields to be simultaneously and consistently interpreted.

## 2 Hyperspectral image processing

### 2.1 Data acquisition

**Figure 1.**
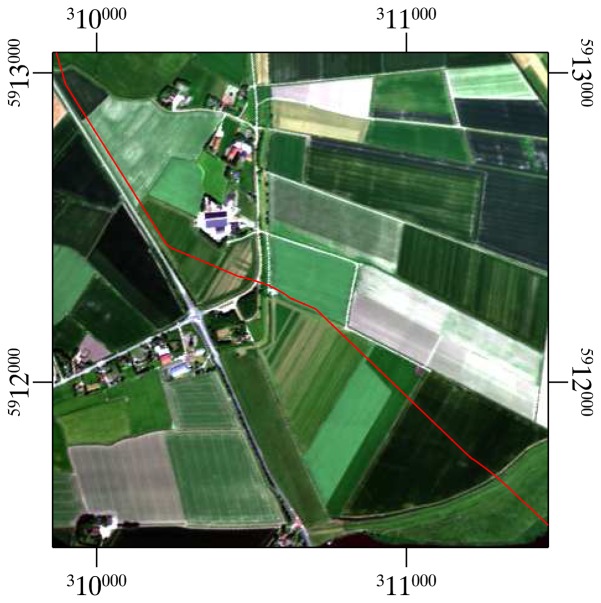
A natural colour composite of the HyMap image, showing the 1 km of the pipeline that has been studied in this research. The location of the pipeline is indicated by the red line.

An overflight with a HyMap airborne imaging spectrometer [15] took place on the 19*^th^* of June 2005. This sensor covers a wavelength region of 436–2485 nm with a nominal bandwidth of 15–20 nm. For this overflight, the altitude was approximately 3000 m which resulted in a 4×4 m pixel size. The imagery was pre-processed by the German Aerospace Center (DLR) in Oberpfaffenhofen, which included an atmospheric correction using ATCOR4 [16] and a geometric correction using PARGE [17]. The HyMap dataset was delivered in reflectance values and comprised two scenes that cover the entire 21 km length of the pipeline. In this paper, we had spatially subset the image to show an area encompassing 1 km length of the pipeline (figure 1).

A field campaign was carried out on the day of the overflight. Reference measurements of bright (brick street) and a dark (canal water) homogeneous surfaces were used for visual inspection of the atmospheric correction. Fieldspectra were acquired with an Analytical Spectral Devices (ASD) FieldSpec Fr Pro instrument. This portable field spectrometer covers a wavelength range of 350–2500 nm with a 1 nm sampling interval and a nominal 3 nm bandwidth. The standard 25-degree Field-Of-View (FOV) bare fiber cable was used without any foreoptics. A similar spectrometer setup was used for measuring vegetation spectra in the field, with the addition of a ’contact probe’ with an internal light source [9].

### 2.2 Calculating vegetation indices

**Figure 3.**
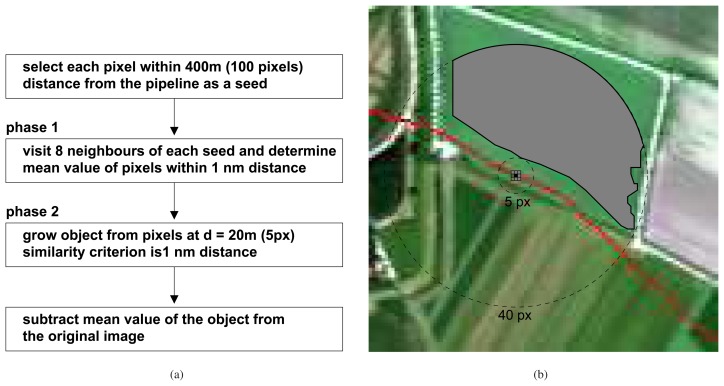
A flow diagram (a) and a schematic spatial overview (b) of pixel normalization using a circular region growing algorithm. The seed (center pixel, in black) is ignored in calculations as it may have an extreme value due to pollution. Instead, 8 neighboring pixels are evaluated and averaged to a reference value if they fall within 1 nm difference of the value of the seed's value. In this example, 6 out of 8 neighboring pixels have been selected to create the reference value. This reference value is then compared with pixels that are between 5 and 40 pixels distance from the central pixel. The pixels that are found in this area and are within 1 nm of the reference value are averaged to become the background value of a field.

The processing of the HyMap imagery initiated with masking those pixels that did not have a dense vegetation cover. Dense vegetation is here defined as more than 70% typical coverage of the ground. Vegetation with a lower density was not used in this study as the effect of soil on the vegetation index was not known. Masking was done by calculating the Normalized Difference Vegetation Index (NDVI) [18] and thresholding the NDVI image at 0.6 after comparison with *a priori* knowledge on land cover. The NDVI image is shown in Figure 3(a).

Reflectance properties of vegetation in the visible part of the spectrum are dominated by the absorption properties of photosynthetic pigments. Chlorophylls are the most important pigments and have their maximum absorbtion in the red light (0.66 and 0.64 *μ*m for chlorophyll a and chlorophyll b, respectively). Vegetation stress causes structural damage to plant cells, which reduces chlorophyll concentration. A decline in chlorophyll concentration produces the following spectral responses: (1) a decrease in the height of the infrared shoulder; (2) a decrease in the maximum absorption; and (3) a shift in the position of the red edge (steep slope between red and near-infrared wavelengths around 0.7 *μ*m) towards shorter wavelengths.

Vegetation indices that had been tested with field spectral measurements [9] include NDVI, Carter stress indices [19], Yellowness Index [20] and red edge position [14]. Although these indices were found to give similar results, the red edge position gave most contrast between polluted and non-polluted fields. With respect to the different spectral resolution of field and image spectra, the red edge calculation after [14] was chosen as it is based on only four spectral bands and not on the entire wavelength range that covers red and NIR. Figure 3(b) shows the red edge values for the study area. The variation in red edge values shows variation between different fields rather than variation within a field, or, different types of vegetation rather than vegetation stress. A normalization procedure is necessary to enhance the intra-field variations.

### 2.3 Normalization procedure

**Figure 4.**
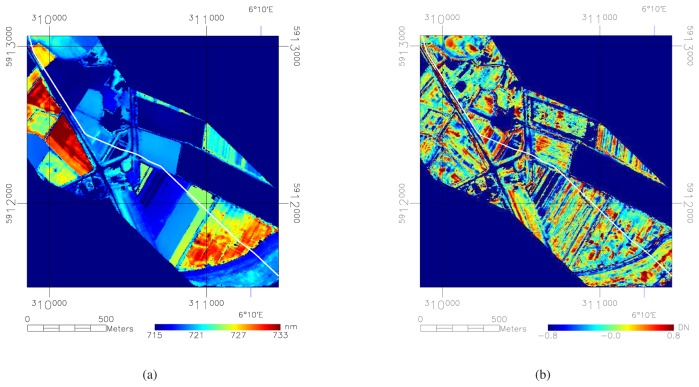
(a) shows the background red edge values for each pixel. The values in each field are homogeneous while boundaries between these fields are crisp. This shows that the region-growing approach can calculate the average red edge value within a field without crossing fields that have a different spectral signature. (b) shows the normalized red edge values for each pixel. This image shows high-frequency changes within different fields while contrasts between different fields are minimized. The location of the pipeline is indicated by the white line.

The normalization procedure (Figure 4(a) was carried out on pixels that fall within 100 pixels distance (approximately 400 m) either side of the pipeline to reduce computation time. The normalization subtracts the average value of a selected region of pixels from each image pixel. These regions were created for each image pixel in a region-growing procedure [21], an approach that is commonly used in image segmentation. The regions were limited to grow to a maximum distance of 40 pixels (approximately 160 m) from a seed (center pixel).

In the first phase, pixels that directly neighbor the seed and have a red edge value within 1 nm distance of the seed's red edge value are selected. The selected pixels, excluding the seed, are averaged, creating a reference value for the object that will grow in the second phase. The similarity criterion of 1 nm is based on field spectrometer measurements, where it was found that anomalous red edge values within a field are in the order of magnitude of 1–2 nm [9].

**Figure 5.**
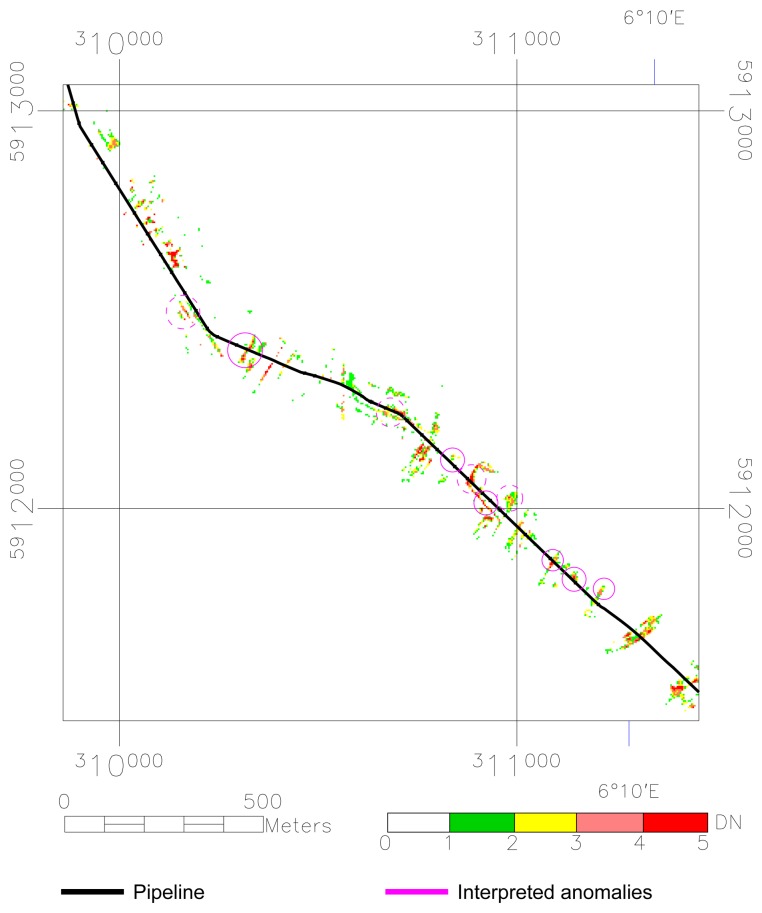
The weighted anomalies on the pipeline. White indicates areas where no anomaly has been found and are interpreted to represent the normal (background) state of vegetation. The pixels with a yellow, orange or red shade indicate areas that are interpreted as having vegetation stress.

In the second phase, the object starts to grow at a ring at 5 pixels distance from the seed. Pixels within 5 pixels distance (approximately 20 m) of the pipeline are excluded to prevent possible anomalous pixels close to the pipeline to influence the average red edge value. The object can continue to grow up to a maximum distance of 40 pixels. For growing, a similarity criterion of 1 nm difference relative to the reference value obtained in the first phase is used. Figure 4(b) shows the distance criteria set in this approach and the effect the region growing procedure has on the selection of pixels. Once the object stops growing, the average red edge value of the object is saved in a new image on the coordinates of the seed (Figure 5(a)). Finally, the image with average red edge values is subtracted from the image with original red edge values (Figure 5(b)).

After the normalization, the image pixels are scaled between -1 and +1 with respect to the background value (Figure 5(a)) determined in the region growing procedure. Values close to -1 mean that the red edge is low with respect to the other pixels in the same field, and *vice versa*. It can be observed that the pattern of red edge values is scattered and that separate fields are no longer distinguishable. This indicates that the normalization procedure has neutralized the influence of spectrally different vegetation types.

### 2.4 Interpretation of the normalized image

Studies of natural and manmade hydrocarbon seepages have shown that the influence of gaseous hydrocarbons leaking from a point source have a horizontal extent of approximately 4 m in sandy soils [22, 23] and 1 m in clayey soils [13]. The influence of liquid hydrocarbons in a clayey soil is therefore not expected to exceed a 4 m distance and to be seen in the HyMap image as subtle spectral anomalies in the pixels that cover or directly neighbor a leak, i.e. the pipeline.

It is evident that not all anomalous vegetation is a result of environmental pollution. Many anomalies occur close to boundaries of fields or are related to in-field management practices such as worked tracks and fertilization. However, by *a priori* knowledge of the location of the pipeline and by using the expected shape and size of anomalies, many anomalies can be ignored, leaving only the anomalies that fulfill the defined pattern of anomalies for interpretation. Anomalies further away from the pipeline are less likely to be caused by processes related to the pipeline. Every pixel was weighted with respect to its distance from the pipeline. First, positive anomalies have been masked out while negative anomalies have been rescaled to an index between 0 and 5 DN that indicates anomaly strength in 5 stages. The weighing was done for pixels between 5 and 30 pixels distance from the pipeline, following equation *V_weighted_* = *V_orig_* − (*distance* − 5)/5, where *V* is the anomaly index and *distance* is distance to the pipeline in pixels. The result can be seen in figure 5.

This still leaves anomalies that are not related to the pipeline. We have asked 4 image interpretation experts to interpret the derived anomalies in terms of potential leakages from a hardcopy print of the processed image. Each anomaly was assessed based on spatial (relative spatial occurrence of anomalies with respect to the whole field) and spectral criteria. The experts had to seek causes for anomalies such as shade thrown by high vegetation, traces of agricultural activities and other human influences. If another cause could not be found, an anomaly was marked as belonging to the pipeline. The anomalies were subsequently assigned into one of four classes; “not”, “possible”, “likely” and “very likely” related to pipeline leakage, by combining the scores of the 4 experts. This resulted in a final classification of the image, based on the combined expert opinion for each separate anomaly, into green, yellow, orange and red. We choose specifically not to use the geochemical ground validation data to train our interpretation. It is very likely that by incorporating this information in future developments of the interpretation algorithms our analysis can be improved. Figure 5 shows the interpreted result after the weighing process and expert analysis. After clustering the anomalies, a total of 28 anomalous regions were identified for the whole pipeline.

## 3 Validation with geochemical measurements

None of the geochemical measurements was used to train the data, so all reference data from the field was available for validation. A total of 38 drilling locations have been used for validation. Table 1 shows the outcome of this comparison. From 30 points where no anomalies had been detected by hyperspectral remote sensing (class “clean”), 2 actually did contain pollution. Anomalies that were detected with hyperspectral remote sensing (classes “possible”, “likely” and “very likely”), were all confirmed by geochemical measurements. Two drilling locations that are classified as neutral in the geochemical data showed mixed interpretations within the identified anomalous regions. This could be due to the strong lateral variations that occur in the clayey soils in the area.

## 4 Discussion and conclusions

Reflectance spectroscopy proved to be a non-destructive tool for identification of anomalous spectral features in vegetation that result from benzene leakage from an underground pipeline [9]. The extension of this result to airborne imaging spectroscopy was a logical step, but introduced the problem on how to analyze and compare various fields with different vegetation cover and state in an automated process. In this paper, we developed a method for image normalization which visualized in-field variations rather than variation between fields. In the intermediate steps of the image processing, one can clearly observe the functionality of the algorithm to derive so-called background values for each separate field in the image.

The normalization procedure resulted in clustering of anomalies in the image. Some of these clusters occurred relatively far away from the pipeline and are not likely to be related to pipeline leakage. The addition of another spatial criterion, limiting the occurrence of anomalies to the direct environment of the pipeline, resulted in a “cleaned” image. In this cleaned image, only those anomalies that fall within a certain buffer of the pipeline are shown. It is important to realize that not every anomaly is necessarily related to the pipeline. There is natural variance in the vegetation that occasionally might appear as a potential pollution anomaly.

In the expert analysis, we tried to avoid the interpretation of natural variance as pipeline related anomaly. Using spatial (relative spatial occurrence of anomalies with respect to the whole field) and spectral criteria, we attempted to minimize the amount of false anomalies in our interpretation. We chose specifically not to use the geochemical ground validation data to train our interpretation. It is likely that by incorporating this information in future developments of the interpretation algorithms our analysis can be improved. Statistical analysis of spectra acquired at polluted and non-polluted sites may give specific wavelengths that are sensitive to benzene pollution, rather than the red edge indicator which only shows overall vegetation state.

Two areas that had not been noticed in the hyperspectral approach appeared to be polluted in the geochemical data. It is not clear if this is a shortcoming of the hyperspectral method or that limiting factors such as a lack of vegetation on approximately 30% of the fields and the time-span between acquisition of spectral and reference (drilling) data are the cause. As it would be better to overpredict than to underpredict polluted sites, these two areas have to be analysed in further detail to improve hyperspectral detection.

It can be concluded that, though only limited ground truth information was available in the test area, the information available on the entire pipeline trajectory showed a correlation between geochemically detected pollution and spectrally identified anomalous regions.

## Figures and Tables

**Figure 2.**
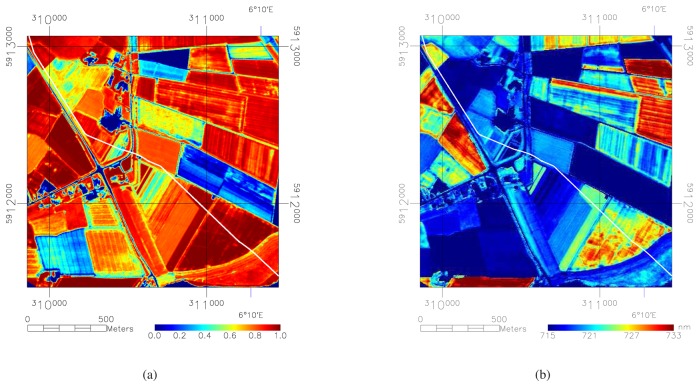
(a) shows the Normalized Difference Vegetation Index (NDVI) values calculated for the test area. High values indicate a dense vegetation cover while low values indicate a lack of vegetation. (b) shows the red edge position calculated for the test area. Lower wavelength values for the red edge position indicate relative vegetation stress. Pixels without sufficient vegetation cover were masked out by an NDVI threshold of 0.6. The location of the pipeline is indicated by the white line.

**Table 1.** Validation of the anomalies detected by hyperspectral remote sensing. 30 clean sites were sampled. Only 8, out of a total of 28, anomalous locations were sampled by drilling. Anomalous locations are divided into three categories; possible (2 counts from the experts), likely (3 counts from the experts), and most likely (all experts marked this location, 4 counts). The drilled locations are classified into polluted, neutral or clean. Neutral locations did not show conclusive evidence for being clean or polluted, they were either around environmental threshold values or gave mixed polluted and clean results.

*Spectral Anomaly*	*No. detected*	*Clean*	*Neutral*	*Polluted*	*Total*
clean	x	28	0	2	30
possible	5	0	0	2	2
likely	11	0	1	2	3
most likely	12	0	1	2	3
